# Flux Analysis of the *Trypanosoma brucei* Glycolysis Based on a Multiobjective-Criteria Bioinformatic Approach

**DOI:** 10.1155/2012/159423

**Published:** 2012-10-13

**Authors:** Amine Ghozlane, Frédéric Bringaud, Hayssam Soueidan, Isabelle Dutour, Fabien Jourdan, Patricia Thébault

**Affiliations:** ^1^Laboratoire Bordelais de Recherche en Informatique, UMR CNRS 5800, Université Bordeaux, 351 Cours de la Libération, 33405 Talence Cedex, France; ^2^Centre de Bioinformatique de Bordeaux, Université Bordeaux Segalen, 142 Rue Léo Saignat, 33076 Bordeaux Cedex, France; ^3^Centre de Résonance Magnétique des Systèmes Biologiques, UMR 5536, Université Bordeaux Segalen, CNRS, 146 rue Léo Saignat, 33076 Bordeaux, France; ^4^The Netherlands Cancer Institute, Plesmanlaan 121, 1066 CX Amsterdam, The Netherlands; ^5^Institut National de Recherche en Agronomie, UMR 1331 TOXALIM, 180 Chemin de Tournefeuille, 31027 Toulouse, France

## Abstract

*Trypanosoma brucei* is a protozoan parasite of major of interest in discovering new genes for drug targets. This parasite alternates its life cycle between the mammal host(s) (bloodstream form) and the insect vector (procyclic form), with two divergent glucose metabolism amenable to *in vitro* culture. While the metabolic network of the bloodstream forms has been well characterized, the flux distribution between the different branches of the glucose metabolic network in the procyclic form has not been addressed so far. We present a computational analysis (called Metaboflux) that exploits the metabolic topology of the procyclic form, and allows the incorporation of multipurpose experimental data to increase the biological relevance of the model. The alternatives resulting from the structural complexity of networks are formulated as an optimization problem solved by a metaheuristic where experimental data are modeled in a multiobjective function. Our results show that the current metabolic model is in agreement with experimental data and confirms the observed high metabolic flexibility of glucose metabolism. In addition, Metaboflux offers a rational explanation for the high flexibility in the ratio between final products from glucose metabolism, thsat is, flux redistribution through the malic enzyme steps.

## 1. Introduction

Trypanosomes are unicellular protozoa that are ubiquitous parasites of higher eukaryotes, including insects, plants, and mammals. Among the numerous species belonging to the trypanosomatid family, *Trypanosoma brucei*, *Trypanosoma cruzi, *and *Leishmania* spp. are responsible for Human diseases. Most of these parasites live in more than one host over their life cycle and encounter very different environments, such as insect vectors' gut and vertebrate bloodstream. Consequently, the different parasitic forms have developed distinct morphologies and metabolisms. 

**Figure 1:**
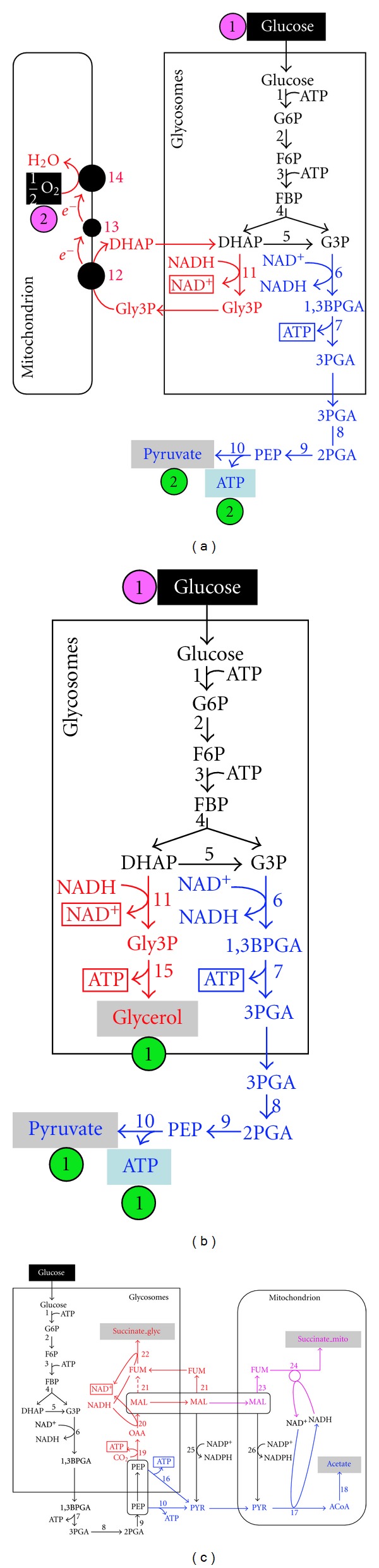
Metabolic network of glucose degradation for the bloodstream and procyclic forms of *T. brucei*.

We will consider here *T. brucei*, which belongs to the group of parasites responsible for sleeping sickness in Africa.* T. brucei* belongs to the only group of organisms that performs glycolysis in a peroxisome-like organelle, called glycosome [1]. It is widely considered that this compartmentalized glycolysis requires impermeability of glycosomal membrane to cofactors, such as NAD(P)^+^ and NAD(P)H, and nucleotides (ATP, ADP, etc.) [2]. As a consequence, the intraglycosomal NAD^+^/NADH and ATP/ADP balances need to be maintained, which implies that each NAD^+^ or ATP molecules consumed during the first glycolytic steps have to be regenerated inside the organelle (see Figure 1).

Panels (a) and 1(b) correspond to the metabolic model of the bloodstream forms of *T. brucei* (BSF) in the aerobic and anaerobic conditions, respectively. Panel (c) represents the metabolic model for the procyclic form grown in glucose-rich medium. For both forms, the major part of the glycolytic pathway is compartmentalized in glycosomes (peroxisome-like organelles). Excreted end-products from glucose metabolism are in red, green, or purple characters on a grey rectangle as background. In Panels (a) and (b), metabolic branches consuming and regenerating NAD^+^ are in blue and red, respectively, while the color code in Panel (c) is blue, red, and purple for the acetate, glycosomal succinate, and mitochondrial succinate branches, respectively. NAD^+^ and ATP molecules are underlined, when consumed in the glycosomes, and boxed, when produced in the glycosomes. In aerobic conditions, BSF converts one molecule of glucose into two molecules of pyruvate with consumption of one molecule of dioxygen (Panel (a)) and net production of two molecules of ATP, while in anaerobic conditions one molecule of pyruvate, glycerol, and ATP is produced per molecule of glucose consumed (Panel (b)). Abbreviations: 1,3BPGA, 1,3-bisphosphoglycerate; DHAP, dihydroxyacetone phosphate; FBP, fructose 1,6-bisphosphate; FUM, fumarate; Gly3P, glycerol 3-phosphate; G3P, glyceraldehyde 3-phosphate; MAL, malate; OAA, oxaloacetate; PEP, phospho*enol*pyruvate; PYR, pyruvate; SUC, succinate. Individual enzymes included in the model are 1, hexokinase: 2, glucose-6-phosphate isomerase; 3, phosphofructokinase; 4, aldolase; 5, triose-phosphate isomerase; 6, glyceraldehyde-3-phosphate dehydrogenase; 7, phosphoglycerate kinase; 8, phosphoglycerate mutase; 9, enolase; 10, pyruvate kinase; 11, glycosomal glyceraldehyde-3-phosphate dehydrogenase; 12, FAD-dependent glycerol-3-phosphate dehydrogenase; 13, ubiquinone; 14, SHAM-sensitive alternative oxidase; 15, glycerol kinase; 16, pyruvate phosphate dikinase; 17, pyruvate dehydrogenase complex; 18, acetate:succinate CoA-transferase and acetyl-CoA thioesterase; 19, phospho*enol*pyruvate carboxykinase; 20, glycosomal malate dehydrogenase; 21, cytosolic (and glycosomal) fumarase; 22, glycosomal NADH-dependent fumarate reductase; 23, mitochondrial fumarase; 24, mitochondrial NADH-dependent fumarate reductase; 25, cytosolic malic enzyme; 26, mitochondrial malic enzyme.

In the mammalian host, the bloodstream forms of *T. brucei* (BSF) develop a very simple and well-known glucose-based energy metabolism, with glucose being converted into the pyruvate, which is the only end product excreted in the presence of oxygen (Figure 1(a)). In aerobiosis, equimolar amounts of pyruvate and glycerol are excreted from glucose metabolism (Figure 1(b)). In both conditions, all ATP required for the parasite development is produced in by the cytosolic pyruvate kinase (step 10 in Figure 1). 

In contrast, the procyclic form of *T. brucei* (PF), which evolves in the midgut of the insect vector (tsetse fly), develops a more complex branched energy metabolism. When grown in standard rich medium, PF primarily uses glucose to provide the cell with carbon and ATP. In the course of glycolysis, phospho*enol*pyruvate (PEP) is produced in the cytosol, where it is located at a branching point (Figure 1(c)). It can be converted into pyruvate, which enters the mitochondrion to produce acetate [3, 4]. PEP can also reenter the glycosomes to be converted to succinate in either the glycosomes or the mitochondrion [5, 6]. Although the topology of the glucose metabolism network is known for the procyclic form, the flux distribution between the different branches of the network has not been addressed so far.

The main objective of this paper is to propose a bioinformatics analysis, integrating multipurposed experimental data, to investigate the flux distribution in the main branches of glucose metabolism of the PF trypanosomes. To address this question, we developed a model based on (i) the published topology of the metabolic network [7, 8], (ii) the maintenance of the glycosomal redox (NAD^+^/NADH) and (ATP/ADP) balances, with no exchange of these cofactors with other subcellular compartments [7], and (iii) experimental data. 

## 2. Related Work

In the last decade, high-throughput technologies had been developed to monitor organism responses to various environmental perturbations. At the same, time many advances in bioinformatics have been made to mine these data. In particular, methods have been designed to investigate the plasticity of biological processes. To conduct such analyses, the level of abstraction of models can range from global to local and from static to dynamic, according to the biological data available (for a review, see [9]). A biological system can be modeled by a set of interconnected reactions allowing fluxes of chemical compounds (metabolites). When the organism is exposed to environmental changes, these fluxes are adjusted to preserve the homeostasis of the metabolism and to optimize biological functions such as growth rate. Several bioinformatics approaches address the problem of estimating the flux distributions accompanying these metabolic perturbations. The quality of these computed flux distributions is critical since it strongly affects the ability of subsequent *in silico* simulations to fit and predict physiological observations. Moreover, the integration of experimental data in such models helps in producing more realistic *in silico* description of biological systems.

One of the most popular formalism for modeling metabolism relies on constraint-based methods such as Flux Balance Analysis (FBA) [10, 11] that are designed to find an optimal flux distribution given a specific objective function (e.g., growth or ATP production). This mathematical framework is based on the stoichiometry of reactions, which is analyzed using linear programming to optimize fluxes toward an objective function. The resulting solution space defines all of the possible metabolic behavior of the cell under a given set of conditions, and the addition of constraints help to predict achievable cellular functions that reflect thermodynamic, kinetic, or biochemical knowledge (for a review see [12]).

To better mimic the *in vivo* system, considerable attention has been directed in recent years towards the development of variants of FBA to (1) explore differently the space solutions or/and (2) integrate experimental data.

The Optimal Metabolic Network Identification [13] and Regulatory On/Off minimization [14] methods suggest adapting the network flux structure to observed data, while the Minimization Of Metabolic Adjustment [15] approach searches for suboptimal, but more realistic solutions. To add flexibility to FBA prediction, Flux Variability Analysis (FVA) [16] proposes a feasible range of fluxes that satisfies the objective function corresponding to different genetic states. To limit the allowable functional behavior of networks, Energy Balance Analysis integrates additional thermodynamic constraints that describe energy balance and eliminate thermodynamically infeasible optima [17]. Other efforts have focused on the exploitation of integrative methods for improving the prediction of metabolic flux distributions. Notably, integrative Omics-Metabolic Analysis [18] and integrated Flux Balance Analysis [19] quantitatively integrate proteomic and metabolomic data with genome-scale models to predict metabolic flux distributions. These methods allow for the integration of kinetics models, when available, and formulate a set of differential equations to describe the dynamics of metabolite concentrations.

However, none of the previous methods account for a multiobjective function, which is crucial since eukaryotic cells perform multiple metabolic functions. Therefore, before simulating such metabolic models, integrating biological relevant knowledge as different and multiple objectives need to be implemented. In practice, the definition of an appropriate framework is a difficult task in increasing the complexity of the flux prediction problem.

Moreover, the identification of a physiologically realistic objective function remains challenging [20] since it strongly constrains the predictive quality of the flux distribution. While it is commonly accepted to use the maximization of the biomass yield for bacteria, other objective functions may be more appropriate to predict metabolic fluxes in eukaryote cells. For instance, systems are ruled by other type of constraints, such as side compound balancing or sustaining certain metabolite concentrations within the system. Most of these constraints can be experimentally monitored and defined as an objective state.

Combining them with the flux objective function could imply several and sometimes conflicting objectives. For these reasons, recent studies investigated the flux balance problem in a multiobjective perspective [21, 22]. These theoretical articles propose algorithms to infer a flux distribution constrained by one or more target optimal output fluxes. These approaches handle several objectives with different impacts. The first step in these approaches consists of setting the bounds of the solutions space. Next, the function to optimize can be defined as a multiparametric distance that can handle data of different kinds (metabolite biomass, fluxes, etc.). The distance is then optimized by nonlinear or linear methods, adapted for multidimensional data. Finally, the aim is to find a configuration of the system, which is as close as possible to the optimal state, and where no improvement of one objective is possible without negatively impacting another objective (namely, an optimal Pareto solution). Several analyses have been carried out to infer the optimal solution when using 2, 3, or 4-objective combinations to illustrate the benefit of the addition of experimental data to mimic the reality of the cell [22]. Besides the great interest of these articles, they are not related to publicly available bioinformatic softwares.

None of the existing methods offers a qualitative or semiquantitative approach that can account for multiple constraints deduced from experimental data. Prediction of flux distribution should account for properties such as cellular homeostasis where there is no net consumption or production of key intracellular metabolites/cofactors (NAD(P)^+^, NAD(P)H, ATP, ADP, etc.), or metabolic data, such as the ratio of excreted end-products. Therefore, we are particularly interested in a modeling system that does not require kinetic data to predict flux distribution, and that can integrate multipurposed constraints based on the known properties of the metabolic network. 

To achieve this goal, we propose and implement a heuristic algorithm that can compute the optimal flux distribution that fits results deduced from various high throughput approaches (e.g., metabolomics and fluxomics). In particular, the real values of the fluxes of most reactions are unknown and span large intervals. Therefore, the key question that our method addresses is to use experimental observations to identify the set of parameter values for which the network model would exhibit a realistic behavior.

The methodology used in this approach (called *Metaboflux*) combines a metabolic network simulator with a probabilistic metaheuristic [23] to optimize multiple flux objectives under multiple biological constraints. *Metaboflux* is freely available at http://services.cbib.u-bordeaux2.fr/metaboflux/.

## 3. Material and Methods

### 3.1. Biological Model and Data

Glucose metabolism of the BSF and PF trypanosomes differs considerably. The first model, described in the next section, corresponds to the simple and well-known glucose metabolism of BSF, with the flux distribution in the two main branches experimentally validated (Figures 1(a)-1(b)). The second model, describes the more elaborated glucose metabolism in the PF trypanosome, with three interconnected branches (Figure 1(c)). However, flux distribution between these metabolic branches has not been addressed so far.

Our bioinformatic approach will be validated through the first well-known BSF model, before being used to investigate the flux distribution in the main branches of the PF glucose metabolism.

**(A) Trypanosoma brucei Bloodstream Forms (BSF)**

When grown in the presence of oxygen, the BSF convert glucose into pyruvate, the excreted end product, in three subcellular compartments, the glycosomes, the cytosol, and the mitochondrion [7]. For reasons of simplification, the cytosolic and mitochondrial compartments shown in the Figure 1(a) are merged in the model. The first seven glycolytic steps take place in the glycosomes (steps 1–7), while the three other steps leading to pyruvate are cytosolic (steps 8–10). It is to note that one molecule of glucose (hexose) is converted into two molecules of triose phosphate, dihydroxyacetone phosphate (DHAP), and glyceraldehyde 3-phosphate. In the glycosomes, ATP molecules consumed in steps 1 and 3 are regenerated by step 7 and NAD^+^ consumed in step 6 is regenerated inside the organelle by conversion of DHAP into glycerol 3-phosphate (G3P) (step 11). The latter is converted back into DHAP in the mitochondrion (step 12), to form a DHAP/G3P cycle, which transfers electrons to dioxygen to produce H_2_O (steps 12–14). Consequently, two molecules of pyruvate are produced from one molecule of glucose consumed, with a net production of two molecules of ATP in the cytosol by the pyruvate kinase (step 10). In anaerobiosis, electrons cannot be transferred to dioxygen, thus G3P is converted into the excreted end product glycerol, with as a consequence a net production of one molecule of pyruvate and glycerol excreted by molecule of glucose consumed, with a net production of one molecule of ATP (Figure 1(b)).

**(B) Trypanosoma brucei Procyclic Form (PF)**

Energy metabolism has been extensively studied in the PF of *T. brucei *(for a review, see [7, 8]); however, only glucose metabolism is taken into consideration in the model. Conversion of glucose into the excreted end products, succinate and acetate, implies glycosomal, cytosolic, and mitochondrial enzymatic steps [7]. As mentioned for the BSF model, the cytosolic and mitochondrial compartments shown in the Figure 1(c) are also merged in the PF model. The first six glycolytic steps take place in the glycosomes and consume 2 molecules of ATP and 2 molecules of NAD^+^ per molecule of glucose consumed (steps 1–6 in Figure 1), while the three other steps leading to phospho*enol*pyruvate (PEP) are cytosolic (steps 7–9). PEP is located at a key branching point, that is, (i) one branch leads to acetate production in the mitochondrion (steps 10, 17-18) [3, 4] and (ii) the other branch is also branched with succinate being produced in both the glycosomes (steps 19–22) and the mitochondrion (steps 23-24) [24, 25]. The glycosomal succinate branch is critical for glycolysis by regenerating one molecule of ATP (step 19) and up to two molecules of NAD^+^ (steps 20, 22) per molecule of succinate produced. The model also includes the essential cytosolic and mitochondrial malic enzymes (ME, steps 25 and 26, resp.) [26], which constitute a bridge between the succinate and acetate branches. It is also important to mention that (i) the tricarboxylic acid cycle is not functioning as a cycle in the PF trypanosomes and, most of acetyl-CoA produced from glucose is converted into acetate [27], (ii) most ATP is produced by substrate level phosphorylation from glucose, with a nonessential contribution of the mitochondrial F_0_/F_1_-ATP synthase for ATP production [4, 24, 26, 28], (iii) NADH molecules produced in the mitochondrion by the pyruvate dehydrogenase complex (step 17) are regenerated by the mitochondrial fumarate reductase (step 24), implying that the respiratory chain activity is not required to maintain the mitochondrial redox balance of glucose metabolism [6]. As a consequence, the mitochondrial tricarboxylic acid cycle, respiratory chain, and F_0_/F_1_-ATP synthase are not included in our model.

### 3.2. Metaboflux

We analyzed the behavior of both the bloodstream and procyclic form of *T. brucei* by semiquantitative modeling and simulation with stochastic Petri Nets (PN). We describe in this section the details of the design of Metaboflux (see Figure 3). Metaboflux is a new framework for the simulation and for the estimation of the parameters of PN with stochastic immediate transitions that we called Fluxes Petri Net (FPN). We first describe the formal definition of the PN variant we use. We then provide a simulation algorithm to generate sequences of markings from a model. We then outline an optimization procedure to estimate the parameters of a model so as to satisfy constraints derived from experimental data. We finally present an alternative and faster and approximate simulation algorithm that we used to approximate the behavior of a model during the optimization steps. 

#### 3.2.1. PN Formalism

The PN formalism gives an interesting framework for simulating biological systems [29] and has been largely employed in the last decade to describe biological networks (for a review see [30]). 

A PN (also called Place Transition net) is a graph-based model that can describe the dynamics of a system as a set of sequences of discrete configurations. A PN is a bipartite, directed and labeled graph. Nodes in a PN can either be a place ﻿*p* ∈ *P*﻿ or a transition ∈*T*﻿. In the case of metabolic networks, places of a PN represent the metabolites and transitions represent enzymatic reactions, labeled by the enzyme catalyzing the reaction. Each place contains a countable (i.e., positive integer) set of tokens, representing the quantity of the corresponding metabolite. The number of tokens *i* : *P* → *ℕ*﻿ in every place is called a marking of the net and represents its current state. Traditionally, the number of tokens in a place *p* is indicated by #(*p*, *i*) or #(*p*)﻿ when the marking is understood. Edges in a PN are only allowed between a place and a transition or vice versa, but never between two places or two transitions. When an edge connects a place and a transition, the edge is called an input arc for the transition. When an edge connects a transition and a place, the edge is called an output arc for the transition. 

A transition is enabled and can fire when all its conditions are fulfilled, namely, if all its input places contain enough tokens. When a transition is enabled, selected, and fired, tokens from its input places are consumed, and tokens are added to its output places. Every edge in a PN is labeled with a multiplicity corresponding to the stoichiometry of the reaction. More formally, the multiplicity (*p*, *t*) ∈ ℝ of an input arc (*p*, *t*) is a positive integer value indicating the number of tokens that must be present in the place *p*﻿ for the transition *t*﻿ to be enabled. The multiplicity ﻿*m*(*t*, *p*) ∈ ℝ﻿ of an output arc ﻿(*p*, *t*)﻿ is a positive integer value indicating the number of tokens that will be added to the place﻿ *p* if the transition *t* fires.

**Figure 2:**
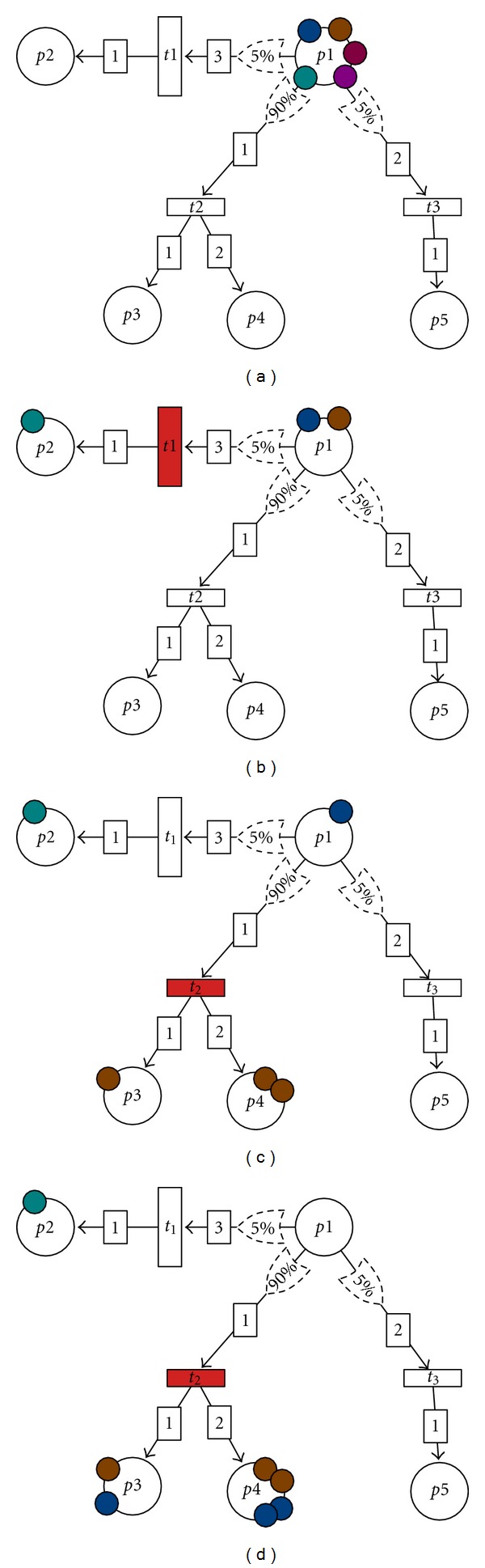
Illustration of the dynamics of a Flux Petri Net (FPN). (a) depicts the initial state of the FPN, while (b)–(d) depict its states after three transitions have been fired. This FPN comprises five places (*p*1–*p*5) and three transitions (*t*1–*t*3). The transition *t*1 consumes three tokens of *p*1, produces one token of *p*2, and has an associated flux distribution of 5%. Similarly, transition *t*2 consumes one token of *p*1, produces one token of *p*3, two tokens of *p*4, and has an associated flux of 90%. In the initial state (a), the place *p*1 contains five tokens, colored differently to be distinguishable. Since *t*1 requires three tokens of *p*1, *t*2 requires one token of *p*1, *t*3 two tokens of *p*1; that *p*1 contains five tokens; the three transitions *t*1, *t*2, and *t*3 are enabled in this configuration. The corresponding probabilities for each transition are *P*(*t*1) = 5/(5 + 90 + 5) = 0.05,  *P*(*t*2) = 90/(5 + 90 + 5) = 0.90  and  *P*(*t*3) = 5/(5 + 90 + 5) = 0.05. Suppose we select *t*1 with probability 0.05, then three tokens of *p*1 are consumed and one token of *p*2 is produced through *t*1. In the second state (b), only *t*2 and *t*3 are enabled, since the required number of tokens for *t*1 in *p*1 is not satisfied. This time, the respective probabilities associated with *t*2 and *t*3 are given by  *P*(*t*2) = 90/(90 + 5) = 0.948,  and *P*(*t*3) = 5/(90 + 5) = 0.052. Suppose *t*2 is selected, it consumes one token of *p*1 and produces one token of *p*3 and two of *p*4. In the third state (c), only transition *t*1 is enabled, and the FPN reaches a state (d) after firing *t*1 where no transition is enabled.

**Figure 3:**
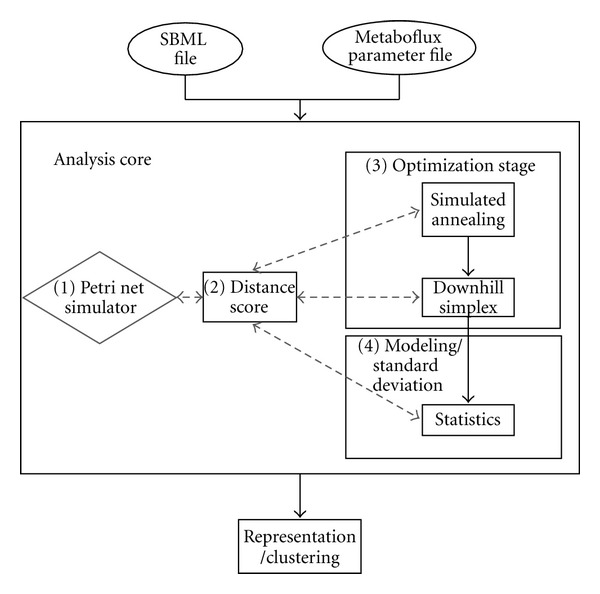
Schematic representation of Metaboflux. analysis procedure Metaboflux takes two files as input, one SBML file that describes the metabolic network topology and an XML file in Metaboflux format indicating simulation parameters. The first step launches N-analysis core by MPI (Message Passing Interface). Each analysis core solves a constrained optimization problem with a simulated annealing and downhill-simplex. These methods suggest candidate optimal fluxes valuation and submit them to the simulator. The simulator estimates the distribution of the final markings according to theses fluxes valuations and returns the corresponding distance score as a result. These steps are repeated until the optimization procedures converge to one solution.

Combining ideas from Generalized Stochastic PN [31] and FBA [10, 11], we extend the PN formalism with fluxes weight. A Flux Petri Net (FPN) is a PN with an additional flux weight labeling of transitions. Formally, a flux weight is a function *w* : *T* → ℝ^+^﻿ that assigns a strictly positive flux value to every transition of an FPN. During simulation, flux weights are used to compute a probability distribution over possible transitions whenever more than one transition is enabled in a given marking. The higher the flux value, the more often this reaction will fire and thus the higher the proportion of metabolites that will go through this reaction. The dynamics of an FPN is illustrated in Figure 2. 

**Figure 4:**
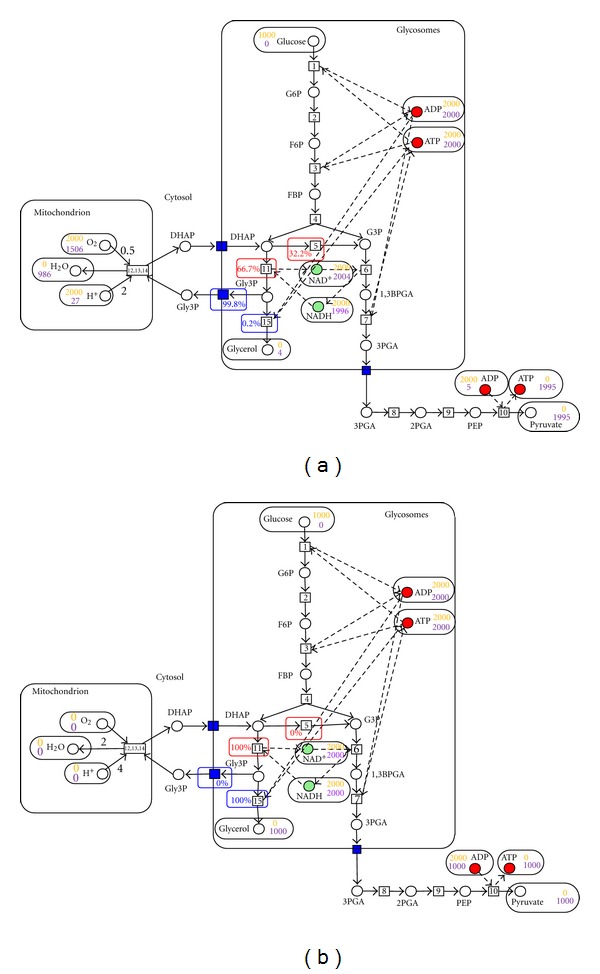
The flux prediction for the well-known BSF model in aerobiosis (a) and anaerobiosis (b). All the considered pathways are located in 3 compartments: mitochondrion, glycosomes, and cytosol. Circles represent metabolites, squares depict reactions, and blue squares are transporters. The stoichiometry is 1 by default, except for H^+^ and H_2_O (the stoichiometries of H^+^, H_2_O and O_2_ were multiplied by 2, resp. 4, 2 and 1). The quantity of input metabolites for the initiation stage of Metaboflux is colored in orange, and the final amount are then colored in purple. The flux percentage at branching points of the metabolic network is given in red for the DHAP node (step 5 versus step 11) and in blue for the Gly3P node (transport_1 versus step 15). The percentage values at branching points have to be considered as a subpart of the 100% of flux given for the branching point where two enzymes use the same metabolite; for instance, glycerol 3-phosphate dehydrogenase (step 11) and triose phosphate isomerase (step 5) use DHAP as substrate.

As an example of FPN, Figure 4(a) depicts the PN model of the bloodstream form of the glucose metabolic network of *T. brucei*. In this figure, places of the net are, as usual, depicted as circles. Transitions are drawn as squares and are labeled with the number of the reaction. Arcs represent input and output arcs with nonzero multiplicities. Flux weights are depicted in the squares bounding transitions. The default multiplicities and flux weights are ﻿1﻿.

To analyze the behavior of a net, we sample one possible sequence of markings with the following stochastic simulator. The simulation starts with a provided initial marking, considered as the current marking for the first iteration of the simulation.

At each iteration of the simulation loop, we update the current marking by selecting and applying an enabled transition. More formally, given a current marking :*P* → *ℕ*﻿, let *E*(*i*) = {*t* ∈ *T* | for  all  *p* ∈ *P*  and  *m*(*p*, *t*) ≤ #(*p*)}﻿ be the set of enabled transitions in the marking *i* ﻿. The probability *w*_*t*_(*i*) that the transition ﻿*t*﻿ will fire in the current marking ﻿*i*﻿ is given by the normalized weight *w*_*t*_(*i*) = *w*(*t*)/∑_*t*′∈*E*(*i*)_ *w*(*t*′). Once a transition *t* is selected by random choice, the next marking *t*′ is defined by subtracting the consumed tokens and adding the produced tokens, that is, #(*p*, *i*′) = #(*p*, *i*) − *m*(*p*, *t*) + *m*(*t*, *p*)﻿. 

This simulation loop is repeated until we reach a user-provided maximal number of iterations or if there are no transition enabled in the current marking. In the case of the FPN models of the BSF and PF of the glucose metabolism of *T. brucei*, we can see that any sequence of markings will eventually reach a final marking where no transitions are enabled. Indeed, these FPNs are both bounded and contain sink places that are not inputs of any transitions. It is straightforward to see that regardless of the initial marking or of the flux weights assigned to transitions (as long as they are non zero), all the tokens in these FPNs will eventually end in one of these sink places. Therefore, after a finite number of iterations, there are no transitions that are enabled and these FPNs reache a final marking. In our simulations and analysis, we are thus guaranteed that these FPN will always reach a final marking.

#### 3.2.2. Parameter Calibration of an FPN under Experimentally Observed Constraints

We now consider the problem of fitting the predictions of an FPN to experimentally observed behavior. We first formalize the fitting problem and then indicate the computational approach we used to solve it as well as an approximate simulation algorithm. Finally, we summarize the experimental data available for the metabolic networks of the BSF and PF.

For the metabolic networks of *T. brucei*, available experimental data provide proportions of the final metabolites and indicate a restored balance between cofactors in the glycosome. These experimental data exclusively concern the state of the end products of the glucose metabolism of the parasite. Consequently, we are interested in the final markings of an FPN and we want them to satisfy the observed balances and proportions of end-products and cofactors. For an FPN, these final markings are completely dependent on a fluxes valuation ﻿$\vec{f}$﻿, that is, the fluxes weights of all its transitions. Therefore, the problem of fitting the predictions of an FPN to the expected behavior is reduced to finding ﻿$\vec{f}$﻿ such that the final markings are in agreement with experimental evidence.

The experimental evidence we account for is of different nature and is formulated using different constraints. Experimental data indicating the expected amount of end-products (e.g., the final quantity of ATP is equal to the initial quantity, or the amount of Acetate excreted in the cytosol) are formulated using the Euclidean distance between final markings and expected quantities. These distances must be minimized. Experimental data indicating that the possible amount of end-products lies within an interval (e.g., the quantity of excreted cytosolic succinate for the PF network) are formulated using logical constraints on the final markings. These constraints must be satisfied. Experimental data indicating expected fluxes values (resp. that flux values lies within an interval) are formulated using Euclidean distances that must be minimized (resp. a constraint on the fluxes valuation that must be satisfied). These distances are summed in a distance function ﻿$D(\vec{f})$ ﻿ that we want to minimize when subject to the conjunction of constraints over markings and fluxes. This function ﻿*D* ﻿ measures the prediction error, that is, the distance between the prediction and the expected quantities. 

For the *T. brucei *BSF model (Figures 1(a) and 1(b)), the modeled constraints are

1. the balance of glycosomic co-factors NAD^+^/NADH and ATP/ADP in the final marking,
2. an expected maximal cytosolic ATP quantity.

For the *T. brucei *PF model (Figure 1(c)), depending on the analysis, the modeled constraints are composed of

1. the balance of glycosomic cofactors NAD^+^/NADH and ATP/ADP in the final markings,
2. the expected proportion of excreted acetate over final acetate and succinates, that is, 50% of end products should consist of acetate,
3. a range of acceptable glycosomal and mitochondrial succinate amount,
4. a minimal flux value for the reaction PEP → OAA (step 19),
5. a minimal flux value for the reaction MAL → PYR (steps 25, 26).

More details on these constraints are provided in the result section. 

To find the flux valuation minimizing the prediction error, we repeatedly evaluate the distance function, and thus perform several simulations of the corresponding FPN until it reaches a final marking. Since our models and simulation algorithm are probabilistic by nature, a single flux valuation can generate multiple final markings (i.e., a distribution over final markings). We thus consider the prediction error associated with a flux valuation to be the prediction error of the average of the final marking of ﻿100﻿ simulation replicates. 

Numerically, we solved this minimization problem by combining two nonlinear, nonderivative based optimization techniques. Optimization problems can be investigated in many scientific areas using metaheuristics such as Genetic algorithms or Simulated Annealing (SA) [23] processes (for a review and comparative performances see [32]). Publications have shown a better efficiency with methods based on SA processes (for instance, [32]) and influenced our choices towards an implementation of SA algorithm. However, thanks to the modular implementation of Metaboflux, a further work might investigate the impact of different solvers according to specific applications in metabolic networks.

In Metaboflux, the first technique we investigated is a global stochastic search based on SA. This method repeatedly evaluates the distance function on fluxes valuation that are chosen randomly. The probability that a candidate flux valuation is accepted as the argument of the minimum of the distance function depends on the corresponding distance value, the difference between the candidate solution and the previous candidate and a gradually decreasing value called temperature. In the first iterations, a high temperature value corresponds to a low probability of acceptance, while in later iterations, a low temperature corresponds to a high probability of acceptance. This saves the method from selecting in the first iterations a local optimum. In this study, we used the GSL [33] implementation of the SA, that we ran with an initial temperature of 10 000. We observed that even with this (relatively) very high temperature, the SA often converged to a local optimum. To avoid this issue, we used a High Performance Computing platform to run 300 individual instances with random starting fluxes valuations. For each of these instances, we use the returned candidate optimum as the starting point of an additional local optimum search based on a Nelder-Mead/downhill-simplex method.

The large amounts of metabolites present in the BSF and PF models imply that each evaluation of ﻿the function *D* requires thousands of iteration of the simulation loop described in the previous section. A single iteration of the simulation loop will select a transition that, once fired, will move a limited number of tokens between places. In the BSF and PF models, the stoichiometries are of ﻿1﻿ except for H^+^ and O_2_ (resp. 2 and 0.5), and thus almost all transitions will move a single token. For a model having ﻿*n*﻿ tokens of glucose as input, and assuming that the places are bounded by *O*(*n*) tokens, the number of iterations of the simulation loop required to reach a final marking is in the order of ﻿*n*∗|*T*|﻿ where |*T*| is the number of transitions in the model. Furthermore, during the minimization procedure, each flux valuation requires 100 simulation replicates. Finally, the prediction error of several thousand different fluxes valuations are evaluated before finding an optimum solution. Due to this huge number of simulation runs to be performed, using the simulation algorithm during the parameter calibration step proved to be computationally infeasible. Instead of simulating the exact semantics of FPNs, we implemented a greedy simulator that approximates their dynamics.

The greedy simulator we used during the parameter calibration step is based on the idea that the intermediate markings are not required for the minimization procedure. At each step of the simulation, the greedy simulator fires *maximally*, that is, moves as much tokens as possible by firing in one step multiple transitions. We identified three situations where multiple transitions can be lumped and fired simultaneously without modifying the final marking. The first situation is when only one transition is enabled. Consider, for example, the situation where tokens are only present in one place that is the input place of a single transition. This is the case for the initial marking where only glucose and cofactors are present. With the greedy simulation, when this single enabled transition is fired, the totality of tokens in the input place is moved to the output place(s). The second situation arises in markings where multiple transitions are enabled but are *compatible*. Two transitions are said to be compatible if they do not share any input place. In this case, the greedy simulator fires maximally all these enabled transition. The third situation arises in markings that enable incompatible transitions. In these situations, we compute for each enabled transitions the maximal number of time it can fire, by accounting for stoichiometries and the number of tokens in its input places. Let *n* be the minimum of these maximal numbers of times each transition can fire. The greedy simulator determines the number of times each transition fires in a single step by sampling a multinomial distribution with *n* trials and whose event probabilities are the (normalized) weights of the enabled transitions. These three heuristics enable a 1000-fold reduction in the number of iterations required to reach a final marking. We verified experimentally that these heuristics yields probabilities of attaining a final marking that are comparable to the exact simulation (according to a T-test, differences of the mean final marking between the exact and approximate simulators were statistically not significant at the 0.05 level).

In the analysis of the BSF model, we considered that a difference of ±9% between the predicted and expected distributions is acceptable. This variability of 9% corresponds to nonsignificant experimental differences. We determined numerically the range of values returned by the distance function when we allow for this variability between the predicted and expected markings. The corresponding distance values range from 0 to 0.2. In the subsequent analysis, we thus used a threshold of 0.2 to identify fluxes valuation yielding simulation results that are “close enough” to the experimental results. 

## 4. Results and Discussions

The aim of this analysis is to predict the flux distribution within glucose metabolic network of *T. brucei *PF. However, before applying our approach to the PF model (4.2), we have evaluated the performance of Metaboflux to estimate the metabolic flux distribution for the well-known metabolic BSF model (4.1).

### 4.1. *Trypanosoma brucei* Bloodstream Forms (BSF)

Many experimental data ranging from kinetics to metabolomics have been obtained for the glucose metabolism of BSF trypanosomes, with the objective to identify the best glycolytic drug targets [34]. Consequently, the glucose metabolic model, essential for the production of ATP and therefore fitness of the parasite, has been well characterized [34, 35]. To validate our computational analysis, we have used Metaboflux to model the complete glucose metabolic network of the parasite grown in aerobic and anaerobic conditions. To initiate the simulator, quantity of tokens has been set to 1000 for the glucose input, to 2000 for an excess of dioxygen (aerobic condition), and for both stocks of correlated molecules for ADP/ATP and NAD^+^/NADH, within the glycosomes. As constraints implemented within the objective function of Metaboflux, the balances for ADP/ATP and NAD^+^/NADH have been constrained within the glycosomes. As described earlier, both conditions are vital for the parasite as none transporter has been identified so far for these metabolites, which implies their sequestration within the glycosomes. Moreover, an additional objective was then specified to maximize the amounts of ATP synthesized by the cytosolic pyruvate kinase (step 10), which is the only known source of ATP in BSF [34, 35].

The simulation and optimization stage of Metaboflux has been carried out and several solutions of flux predictions have been proposed while the maximization of the ATP production was well satisfied. Considering the best solutions, which are identical for this model (even if Metaboflux allows the prediction of alternatives), three main observations can be pointed out. First, only pyruvate is produced in aerobiosis, with two molecules produced per molecules of glucose consumed (the quantity of tokens given by Metaboflux is the double of the glucose input stock), while nearly no glycerol is produced in the glycosomes (Figure 4(a)). However, in anaerobic condition, Metaboflux predicts equimolar production of pyruvate and glycerol (data not shown). Second, Metaboflux predicts that nearly two (1995 in Figure 4(a)) and one (1000 in Figure 4(b)) molecules of ATP are produced per molecule of glucose consumed in aerobic and anaerobic conditions, respectively, which fit perfectly with the BSF model. Third, flux distribution at the two branching points, DHAP and glycerol 3-phosphate (Gly3P), is also consistent with the model. In anaerobic conditions, identical metabolic fluxes are required in the glycerol and pyruvate branches (red and blue in Figure 1(b), resp.) to maintain both ATP/ADP and NAD^+^/NADH glycosomal balances. As a consequence, all of the DHAP molecules needs to be converted to Gly3P and step 5 (conversion of DHAP into G3P) is negligible, as predicted by Metaboflux (Figure 4(b)). Similarly, the Gly3P transporter is not contributing, since Gly3P need to be converted into glycerol to maintain the glycosomal ATP balance (Figure 4(b)). In contrast, the flux distribution at these branching points is different in aerobic conditions. The DHAP/Gly3P cycle implies no glycerol in production with a full contribution of the Gly3P transporter, as predicted by Metaboflux (99.8% of the flux in Figure 4(a)). At the DHAP node, an important part of DHAP is converted into G3P, since DHAP produced from Gly3P (steps 11, 12 in Figure 1(a)) reenters into glycosomes. Altogether, these data are in accordance with the BSF experimental data provided in both incubation conditions and validate Metaboflux as an appropriate tool to study flux distribution in the different branches of glucose metabolism in the PF trypanosomes. 

### 4.2. *Trypanosoma brucei* Procyclic Form (PF)

**Figure 5:**
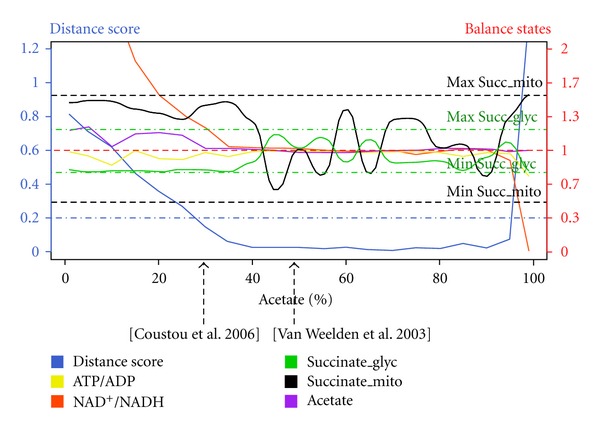
Results for the distance score and balance constraint while varying the Acetate/Succinate proportions.These curves summarize several metabolite proportions as a function of the acetate/succinate proportion. For this experiment, the model was run with 3 constraints (1) The constraints over acetate/succinate proportions ranging from 1 to 99%, with 5% increment, (2) the proportion between the two succinate pools constrained to the ratio 56–86% for succinate_glyc against 14–44% for succinate_mito, and (3) ATP/ADP and NAD^+^/NADH balance being required to be equilibrated. The distance score (blue curve) is a function of the satisfaction of these constraints. When the distance score is less than 0.2, we consider that all the constraints are satisfied. We see that the constraints are satisfied for a proportion of acetate/succinate ranging between 26 and 95%. This corresponds to a predicted flux flexibility of 69%. In this area of flexibility are found the experimental results given by two publications. These experimental observations are indicated by two black dashed arrows.

The purpose of the Metaboflux method developed here is to analyze the extent of the *T. brucei* metabolic flexibility when constraining the system by five different constraints known from experimental data or assumed from the literature, that is, (i) the topology of the metabolic network shown in Figure 1(c), (ii) the ATP molecules consumed in the glycosomal steps 1 and 3 have to be generated by the glycosomal steps 16 and 19, (iii) similarly, the NAD^+^ molecules reduced in the glycosomal step 6 have to be reoxidized in steps 20 and 22, (iv) between 56 and 86% of the total excreted succinate have to be produced in the glycosomes, and (v) conversely, 14 to 44% have to be produced in the mitochondrion. These latter two constraints were deduced from the maximum capacity estimated for both succinate production branches using reverse genetic approaches [6]. After modeling these five constraints in Metaboflux, the optimization was run with fixed proportion of acetate excretion ranging from 0 to 100%, with a 5% increment. We observed that all the constraints were accommodated for acetate proportion (given by the number of acetate tokens under the total number of excreted tokens, that is, succinate plus acetate) ranging between 26 and 95% (distance scores below 0.2) (Figure 5). Figure 5 also shows that all the constraints are accommodated with their score variations. 

The model confirms that the trypanosome glucose metabolism is highly flexible in terms of utilization of the acetate versus succinate branches. Interestingly, the model is also consistent with the experimental data, since the percentage of acetate excreted from glucose metabolism varies between 26 and 80 depending on the analyses [27, 36]. This suggests that a high flexibility of flux distribution between the different branches of the network exists, although tightly constrained by the glycosomal succinate branch to maintain the organelle NAD^+^/NADH and ATP/ADP balances.

**Figure 6:**
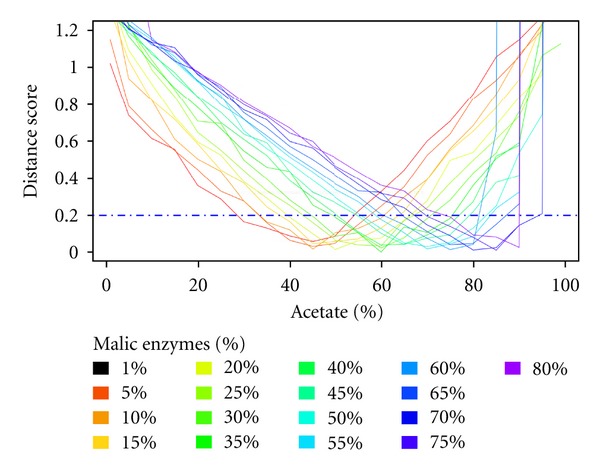
Profile of the distance scores according to the ME flux. This graphic represents the minimal distance score when the acetate proportion, the flux through PK and the flux through ME are constrained. The constraint of acetate/succinate proportion ranges from 1 to 99%, with a 5% increment. In addition to the constraints used in Figure 5, two constraints specify the flux through ME (steps 25-26) and the flux through PK (step 19).

**Figure 7:**
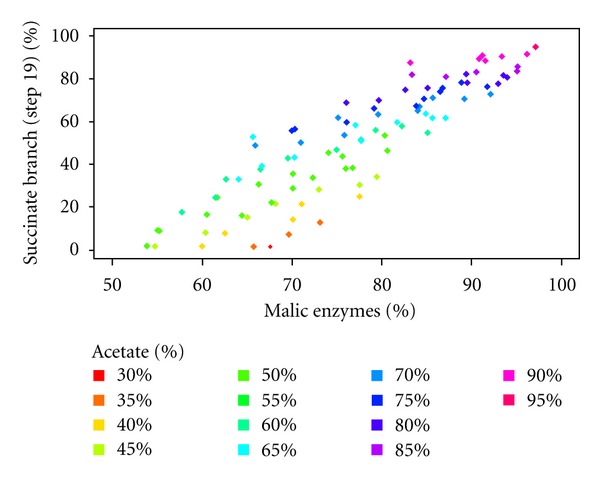
Succinate branch/Malic enzyme. The biological constraints used for the previous analysis (Figure 5) have been also applied here. We represent the flux (%) between the succinate branch (step 19) and the ME. Only the distance points under the 0.2 limit are considered. The flux through the succinate branch increases linearly with the flux through the ME.

To determine possible reasons for this flexibility, we included two additional constraints in the model to adjust the flux through PK and ME (from 1 to 80%, with a 5% increment), thus constituting a bridge between the succinate and acetate branches (steps 25 and 26).  Figure 6 shows only the distance curve for each ME flux according to the proportion of acetate production. It clearly appears that restraining the flux through ME reduces the flexibility of the system. For instance, a 25% flux through ME is compatible with an acetate production (considering the total number of excreted tokens given for acetate and total succinate) ranging between 42 and 69% (Figure 6). This also shows a direct correlation between the flux through ME (between 1 and 70%) and the proportion of acetate production. However, increasing the ME flux by over 70% does not increase the flexibility of the system. The correlation between acetate production and flux distribution through the ME steps, implies that the involvement of the first step of the succinate fermentation branches (step 19) increases proportionally, as observed in Figure 7. Altogether, this analysis suggests that the flexibility of the flux distribution between the succinate and acetate branches is considerably increased by the ME activity, which are essential steps for the viability of the PF trypanosomes [26].

Metaboflux has provided a rational explanation for the high flexibility in the ratio between acetate versus succinate production from glucose metabolism, that is, flux redistribution through ME steps. Interestingly, we previously showed that downregulation of the expression of both ME genes by RNAi is lethal for the PF trypanosomes [26]. Since the main (albeit not the only) role of ME is to provide NADPH required for biosynthetic pathways and to respond to oxidative stresses, one may consider that flux through ME depends on NADPH demand. For instance, under oxidative stress conditions, redistribution of metabolic fluxes through the ME steps, to increase NADPH production in the cytosol and the mitochondrion, would lead to an increase in the acetate/succinate ratio.

## 5. Conclusions

This study presents a bioinformatics analysis of the flux distribution of the BSF and PF *Trypanosoma brucei* glucose metabolism under multiple and varying constraints deduced from experiments. The results suggest that the current definition of the biological model is compatible with all the constraints known from experimental data. Furthermore, our PF model predicts that there is high flexibility in the ratio of acetate to succinate production, which is consistent with data sets of various sources. Notably, our analysis suggests that the malic enzymes are the main support of this flexibility. This predicted property can be confirmed experimentally by metabolic flux analysis. These results have been obtained with the optimization of a multiobjective function that integrates heterogeneous experimental data. In addition, we demonstrate here the great potential of flux analysis to improve the quality of metabolic models. We also demonstrate that combining flux prediction with qualitative constraints derived from experimental data increases the predictive power of *in silico* flux analysis. Our models, simulation and analysis framework, shows great potential and allows for a more realistic investigation of the *T. brucei* metabolism. 

## Figures and Tables

**Figure 8:**
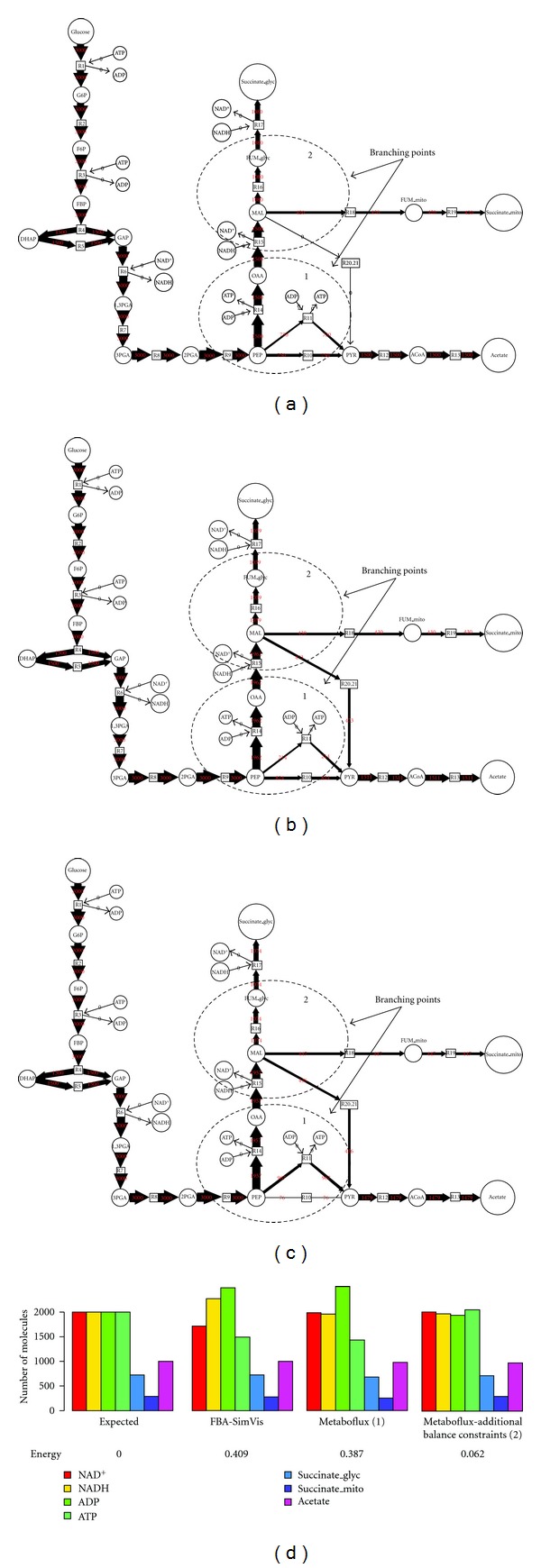
Comparison of the prediction performance between FBA-SimVis and Metaboflux. The flux proportion is given by the edge size, circles stands for metabolites, and square for enzymes. (a) Flux map obtained by FBA-SimVis. (b) Flux map obtained by Metaboflux using a single objective function. (c) Flux map obtained by Metaboflux with additional constraints on balance states and experimental knowledge. (d) Histogram that gives the expected proportion (considering experimental data), and proportions obtained with Metaboflux using the FBA-SimVis flux results as constraints and Metaboflux. The metabolite biomasses were deduced from the number tokens given by Metaboflux. Deduced from empirical measurements, the glucose biomass was set to 1000 tokens and ATP, ADP, NADH, and NAD^+^ were set to 2000. Experimental data given as ratios of excreted metabolites were used to specify expected number of tokens. The balance ratio of ATP, ADP, NADH, and NAD^+^ was specified by an equality equation.

## Appendix

### Comparison of FBA and Metaboflux

FBA and Metaboflux both aim at finding a flux distribution that fits *in vivo* observations as closely as possible. However, Metaboflux goes further by allowing the integration of biological constraints not only on fluxes but also, for instance, on metabolite biomass. In order to measure the benefit of this approach, we carried out a comparative analysis between FBA implemented in FBA-SimVis [37] and Metaboflux. To perform this evaluation, we took time-consumption and the sensitivity of predictions into consideration. Our comparative analysis is based on the metabolic network model deduced from experimental data [7, 8].

Using FBA, we integrated the proportions of acetate and succinate (excreted metabolite) by adding a supplementary reaction to the model (with a specific stoichiometry of 36, 14 and 50) thus linking together the three ways of synthesizing these metabolites. The unique objective function was specified as a biomass production reaction from these three metabolites. With Metaboflux, we run two simulations: (i) as used in FBA, an objective function was used to optimize the biomass production and (ii) a multiobjective function was used to minimize the differences given by the proportion of excreted metabolites known from experimental data. In addition, the balance scales of ADP/ATP and NAD^+^/NADH were constrained within the glycosomes as this condition is required for maintaining the glycolytic flux in trypanosomes. The results of the three flux predictions are given in Figure 8. The flux prediction, using FBA or Metaboflux with a unique objective function, satisfies the constraints on the proportion of final metabolites (specified through the biomass production reaction); however, the balance scales of ATP/ADP and NAD^+^/NADH were not maintained. Using Metaboflux that exploits experimental data in the multiobjective function, the constraints on balance scales and known metabolite proportions were satisfied. Contrary to FBA and Metaboflux with an objective function for the biomass production, fluxes predicted by Metaboflux integrating experimental data are relevant as they obtain the balance scales and the right excreted metabolite proportions. Detailed analysis of the flux predictions on the flux maps (Figures 8(a)–8(c)) shows that the main differences between these methods occur at branching points 1 and 2. Indeed, FBA did not use the path through the ME and distributed the flux equally between pyruvate kinase (step 10) and pyruvate phosphate dikinase (step 16). Metaboflux, on the other hand, considered pyruvate phosphate dikinase, which participates in the maintenance of the ATP/ADP balance. Consequently, 22% of the flux from glycosomal malate dehydrogenase (step 19) goes through the ME to reach the expected acetate/succinate proportion.

This analysis shows the positive contribution of multipurpose experimental data in a metabolic model using Metaboflux. The resulting conflicting constraints are integrated in Metaboflux that can deal with them as our approach is based on a nonlinear optimization. The satisfaction of such constraints is very helpful proposing the development of more realistic models for analysis. FBA is not designed to take this complexity into account and consequently seems to favour the simplest path for its flux prediction.

Metaboflux takes more time compared to FBA-SimVis (319 versus 6 seconds). The difference is mainly due to the time taken by the optimization stage of our method and is correlated to the number of branching points in the network. As the framework of FBA works with a stoichiometric matrix, the complexity of the network slightly affects its running time and such an approach has great application possibilities for exploiting genome-scale metabolic models. Metaboflux is more dependent on the complexity of networks and to deal with this issue, our method has been parallelized. This design is particularly suitable for the new development in computer hardware architectures since *n*-core processors are now available. The parallelization on the different cores will thus make the computation on individual computers faster.
